# Spatial Summation in the Glaucomatous Macula: A Link With Retinal Ganglion Cell Damage

**DOI:** 10.1167/iovs.64.14.36

**Published:** 2023-11-27

**Authors:** Giovanni Montesano, Tony Redmond, Pádraig J. Mulholland, David F. Garway-Heath, Giovanni Ometto, Dario Romano, Federica Antonacci, Lucia Tanga, Carmela Carnevale, Luca M. Rossetti, David P. Crabb, Francesco Oddone

**Affiliations:** 1 City, University of London, Optometry and Visual Sciences, London, United Kingdom; 2 NIHR Biomedical Research Centre, Moorfields Eye Hospital NHS Foundation Trust and UCL Institute of Ophthalmology, London, United Kingdom; 3 School of Optometry and Vision Sciences, Cardiff University, Cardiff, United Kingdom; 4 Centre for Optometry and Vision Science, Biomedical Sciences Research Institute, Ulster University, Coleraine, United Kingdom; 5 ASST Santi Paolo e Carlo, Eye Clinic – University of Milan, Milan, Italy; 6 IRCCS Fondazione Bietti, Rome, Italy

**Keywords:** glaucoma, spatial summation, perimetry, retinal ganglion cells (RGCs), structure-function

## Abstract

**Purpose:**

The purpose of this study was to test whether functional loss in the glaucomatous macula is characterized by an enlargement of Ricco's area (RA) through the application of a computational model linking retinal ganglion cell (RGC) damage to perimetric sensitivity.

**Methods:**

One eye from each of 29 visually healthy subjects <40 years old, 30 patients with glaucoma, and 20 age-similar controls was tested with a 10-2 grid with stimuli of 5 different area sizes. Structural estimates of point-wise RGC density were obtained from optical coherence tomography (OCT) scans. Structural and functional data from the young healthy cohort were used to estimate the parameters of a computational spatial summation model to generate a template. The template was fitted with a Bayesian hierarchical model to estimate the latent RGC density in patients with glaucoma and age-matched controls. We tested two alternative hypotheses: fitting the data by translating the template horizontally (H_1_: change in RA) or vertically (H_2_: loss of sensitivity without a change in RA). Root mean squared error (RMSE) of the model fits to perimetric sensitivity were compared. Ninety-five percent confidence intervals were bootstrapped. The dynamic range of the functional and structural RGC density estimates was denoted by their 1st and 99th percentiles.

**Results:**

The RMSE was 2.09 (95% CI = 1.92–2.26) under H_1_ and 2.49 (95% CI = 2.24–2.72) under H_2_ (*P* < 0.001). The average dynamic range for the structural RGC density estimates was only 11% that of the functional estimates.

**Conclusions:**

Macular sensitivity loss in glaucoma is better described by a model in which RA changes with RGC loss. Structural measurements have limited dynamic range.

Glaucoma is characterized by progressive loss of the visual field (VF) as a consequence of damage to, and death of, retinal ganglion cells (RGCs).^1^^,^^2^ VF damage is usually detected and monitored with standard automated perimetry (SAP), in which circular stimuli of constant area and duration are modulated in luminance on a uniform background at different VF locations. The test aims to estimate, for each location, the stimulus luminance that represents the just noticeable difference from the background luminance. This is expressed as VF sensitivity, where decibel units measure the attenuation of the brightest stimulus (higher decibel [dB] indicating dimmer stimuli). Despite a long-established understanding that perimetric sensitivity is associated with RGC density,^3^^–^^6^ in that they co-vary in disease, such as glaucoma, their exact relationship has proven difficult to elucidate.

Useful insights into the pathophysiology of visual loss in glaucoma can be gathered by studying how perimetric sensitivity changes with stimulus area. For a given duration and background luminance, sensitivity is known to increase with the area of the stimulus (spatial summation).^7^ The change in sensitivity is steeper and directly proportional to the area of the stimulus (complete spatial summation) up to a certain critical area (Ricco's area, or the area of complete spatial summation). After this point, sensitivity still increases with the stimulus area but by a smaller amount (partial summation). Ricco's area is known to enlarge with eccentricity and different stimulating conditions and it has been hypothesized that a critical number of RGCs underlies Ricco's area across different eccentricities,^8^^–^^14^ this varying with adaptation level.^15^ Similar scaling of Ricco's area with RGC density has been hypothesized to hold true with RGC loss in glaucoma.^16^ Redmond et al. demonstrated that Ricco's area is enlarged in glaucoma, which can account for the difference in sensitivity between patients and healthy controls for conventional Goldmann III stimuli.^16^ Antwi-Boasiako et al. showed similar results in nonhuman primates.^17^

The use of computational models has been pivotal to the understanding of these phenomena. Swanson et al.^18^ showed that spatial summation phenomena can be reproduced by a two-stage hierarchical process involving RGC density as well as the spatial tuning of cortical filters, which can be independent of the underlying density of RGCs. Further research by Pan and Swanson suggested that probability summation across RGCs cannot explain spatial summation of perimetric stimuli, whereas it may be explained instead by cortical pooling by multiple spatial mechanisms.^19^ We have recently proposed a computational model able to reproduce the interaction between stimulus area and duration in the response of a synthetic RGC mosaic in healthy observers.^20^ In that work, we also hypothesized, in partial agreement with Swanson et al.,^21^ that the retinal input would determine the selection of different cortical filters, altering spatial summation. We hypothesized that this retinal input could also be altered by a change in the density of RGCs. Under this assumption, we showed that our model would be able to reproduce the results presented by Redmond et al.^16^ in glaucoma.

Glaucoma damage in the macula has been documented extensively in the literature,^22^^,^^23^ but has gained increasing attention in recent years after reports that it can be affected in early disease,^24^^–^^26^ albeit often going undetected clinically until later in the condition,^27^^,^^28^ and that it affects quality of life of patients at all stages of disease.^29^ In the healthy eye, sensitivity measures with the Goldmann III stimulus adopted in SAP (0.43 degrees in diameter) in photopic conditions are determined by complete spatial summation only outside the central 15 degrees.^8^^–^^10^^,^^21^ This means that early macular damage from glaucoma would produce only small changes in SAP sensitivity until a very large proportion of RGCs is lost.^16^^,^^18^^,^^30^^,^^31^ Despite its relevance, only two studies investigated spatial summation in the glaucomatous macula, one in nonhuman primates^17^ and one in patients with glaucoma.^17^^,^^32^ However, they limited their analysis to early damage. Moreover, the investigation in patients with glaucoma ^32^ only correlated sensitivity with coarse RGC count estimates from optical coherence tomography (OCT) imaging, rather than attempting to model the underlying latent process of damage.

In the current study, we wished to test the hypothesis that changes in sensitivity in the macula of patients with glaucoma could be explained by a change in the spatial scale used by the visual system that relates to RGC loss or damage. Here, we perform 5 separate SAP examinations, each with a different fixed-area luminance-modulated stimulus on a 10-2 grid, in eyes with glaucoma with different levels of damage and age-similar healthy control eyes, as well as in young healthy eyes. We then compare our functional RGC density estimates derived from the spatial summation model with structural estimates from high-density OCT scans, to determine the extent to which VF damage can be predicted from clinical measures of tissue loss in the macula.

## Methods

### Study Population

Data were collected in the eye clinic at Santi Paolo e Carlo Hospital – University of Milan, Milan, Italy, and in the glaucoma clinic at IRCCS Fondazione G. B. Bietti, Rome, Italy.

Thirty young healthy participants were recruited among staff and students on a voluntary basis. Inclusion criteria for this cohort were: (1) age between 18 and 40 years; (2) best corrected visual acuity (BCVA) of 0 logMAR or better; (3) intraocular pressure (IOP) < 21 mm Hg; (4) no evidence of ocular disease on preliminary ophthalmoscopic examination; and (5) no history or evidence of systemic disease that might affect the VF or compromise the execution of the test. Individuals were excluded if the macular or optic nerve head (ONH) OCT scans collected for the study showed any signs of ocular disease (details of the imaging and macular testing protocols are reported later). A 24-2 Swedish Interactive Thresholding Algorithm (SITA) VF test was performed for descriptive purposes for the study but was not used to assess inclusion.

Patients with glaucoma and the age-similar healthy participants were recruited on a voluntary basis. Medical charts were screened by clinicians in order to identify potentially eligible candidates. To meet eligibility criteria, patients were required to have a confirmed clinical diagnosis of open angle glaucoma (which could include pseudoexfoliative and pigment dispersion glaucoma), regardless of the integrity of their VF. Eyes with glaucoma were stratified by level of damage according to the mean deviation (MD) value from their most recent reliable (false positive >[FP] < 15%) 24-2 SITA test and classified as early (MD better than −6 dB), moderate (MD between −6 dB and −12 dB), or advanced (MD worse than −12 dB), with the aim of recruiting 10 participants for each class. Other inclusion criteria were: (1) age greater than 18 years; (2) BCVA of 0.2 logMAR or better; and (3) no history or evidence of other ocular or systemic diseases, other than glaucoma, that might affect the VF or compromise the execution of the test. Age-matched controls were recruited among members of staff and patients’ spouses, partners, and relatives. Inclusion criteria were the same as for the healthy young cohort, but with no upward age limit and the requirement for VA to be better than or equal to 0.2 logMAR.

Written informed consent was obtained from all participants. The study adhered to the tenets of the Declaration of Helsinki and was approved by local ethics committees (Comitato Etico Milano Area 1 −code OCU_SSSF; Comitato Etico Centrale IRCCS Lazio N. 90/19/FB).

### Study Protocol

All healthy participants underwent an ophthalmoscopic examination and measurement of their BCVA and IOP (Goldmann Applanation Tonometry) in order to confirm eligibility. Their BCVA was not tested beyond 0 logMAR. BCVA and IOP were not recorded for the study and only used to assess the exclusion criteria. Axial length and corneal curvature were measured with an IOLMaster (Carl Zeiss Meditec, Dublin, CA, USA) and recorded for the study.

Only one eye per participant was included in the study. Where both eyes of healthy controls were eligible, one was chosen arbitrarily by the researcher for testing. In the glaucoma cohort, if the two eyes were classified as having a different stage of glaucoma, one was chosen to populate the severity group, as needed. Otherwise, one was chosen arbitrarily by the researcher.

#### Standard Automated Perimetry

All VF tests were performed with a Humphrey Field Analyzer (HFA; Carl Zeiss Meditec, Dublin, CA, USA). Participants’ near correction was used where required. For young healthy participants, near correction was used according to their preference. All healthy participants underwent a 24-2 SITA Standard test to obtain MD and pattern standard deviation (PSD) values for descriptive purposes.

Separate macular perimetric tests were performed with a 10-2 grid, full-threshold strategy, each with a different Goldmann stimulus diameter (in degrees): G-I (0.10); G-II (0.21); G-III (0.43); G-IV (0.86); and G-V (1.72). The order of these tests was randomized following a computer-generated sequence of tests, one for each subject. For the young healthy cohort, the G-I test was repeated twice, because results with this stimulus were expected to be more variable.^20^ For patients with glaucoma and age matched controls, the G-III test was performed twice instead, to produce a more reliable estimate of the age-corrected sensitivity loss, because normative databases in the HFA are only available for the G-III stimulus. All participants performed a total of six 10-2 SAP tests. Based on previous literature for full-threshold tests,^33^ reliability of the tests was only assessed with the percentage of FP errors (< 33%). For the healthy participants, a limit of 33% on false negative errors was also set. The operator was instructed to carefully monitor the participants and ensure good fixation throughout the test. If unreliable, the test, but not the participant, was excluded from analysis. Fixation losses were not used to determine good fixation because of their poor reliability as a fixation metric.^33^

#### OCT Imaging

Spectral domain OCT (SD-OCT) imaging was performed with a Spectralis SD-OCT (Heidelberg Engineering, Heidelberg, Germany). A circumpapillary retinal nerve fiber layer (cp-RNFL) scan and a high-density macular cube (121 vertical B-scans, 30 × 25 degrees) were acquired. These scans were inspected by an ophthalmologist (the author G.M.) to confirm the absence of any abnormality in the healthy cohorts and of any ocular disease other than glaucoma in the glaucoma cohort. Scans were judged of sufficient quality if all the layers could be clearly identified in the central 15 degrees around the fovea. No scans were removed because of poor quality.

**Figure 1.**
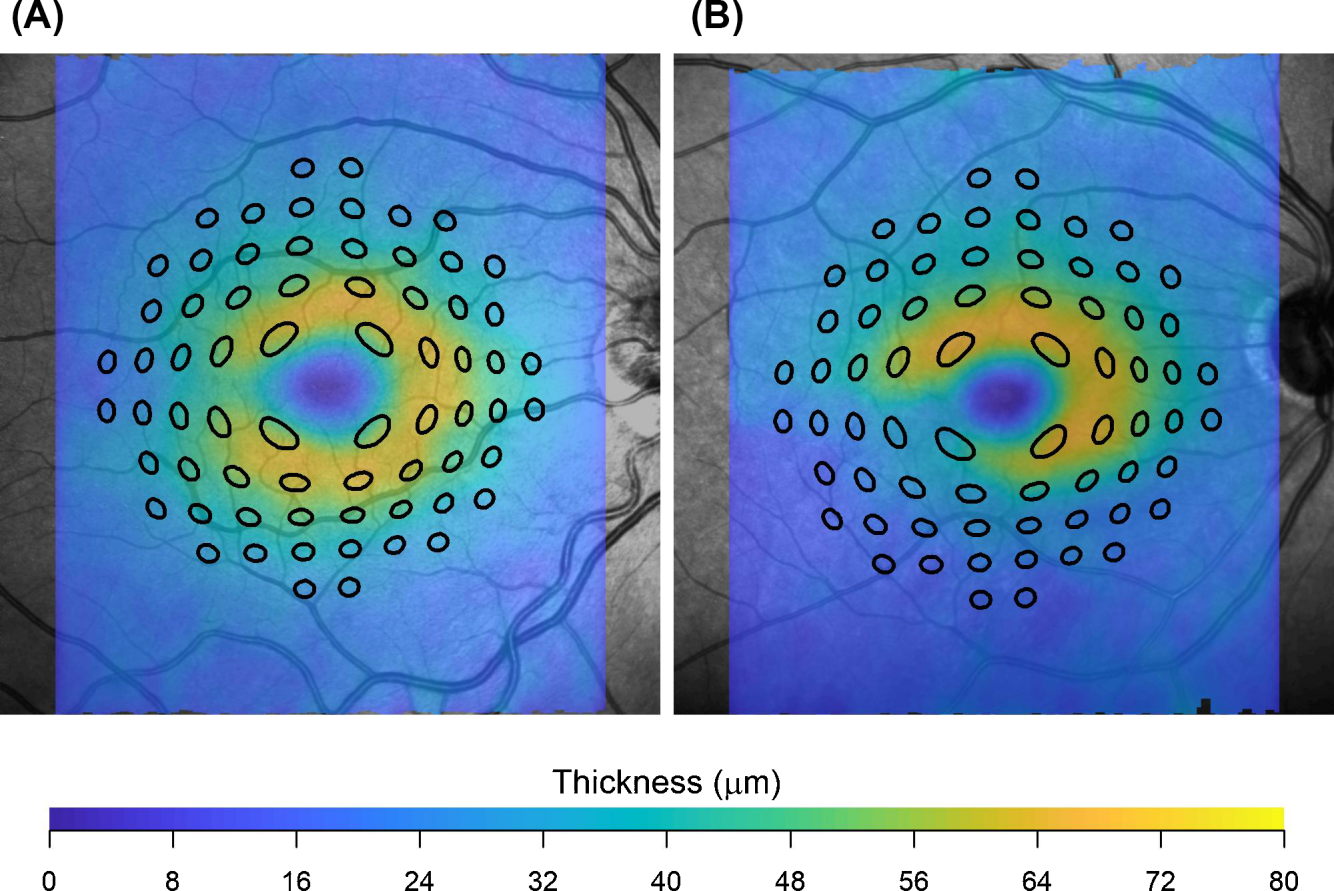
Test locations of the 10-2 grid distorted and displaced to cover the corresponding area on the ganglion cell layer thickness map in a healthy eye (**A**) and an eye with glaucoma (**B**). This example is for a G-V stimulus, for ease of visualization.

Macular volumes were then exported in RAW binary format (.vol) using the Heidelberg Eye Explorer platform and read into R (R Foundation for Statistical Computing, Vienna, Austria). This file contained raw image files and segmentations of retinal layers, including the inner limiting membrane (ILM), Bruch's membrane (BM), the RNFL, and ganglion cell layer (GCL). These segmentations were checked for errors by an ophthalmologist (the author, G.M.) and corrected where needed. Retinal thickness and GCL thickness maps were generated and processed as previously described to obtain localized estimates of the number of RGCs underlying each stimulus area at all locations in the 10-2 grid.^20^^,^^30^^,^^34^ Briefly, the fovea was automatically located via template matching on the retinal thickness map. The GCL thickness map was transformed into an RGC density map with histology data from Curcio and Allen^35^ using a method proposed by Raza and Hood.^36^ This method accounts for eccentricity because the histology-derived volumetric density varies at different positions on the retina. The area covered by the stimuli was displaced and distorted to account for RGC displacement according to a revised version of the model proposed by Drasdo et al.^30^^,^^34^^,^^37^ (Fig. 1). Note that our method for displacement is different from the one used by a similar previous study in the field,^32^ and produces different RGC counts especially in the parafoveal region. However, our method was confirmed to be accurate.^34^^,^^38^ All calculations were performed in visual degrees because we have previously shown that, under a spherical expansion model of the eye, calculations of RGC density in visual degrees are unaffected by axial length.^34^ There is anatomic^34^ and psychophysical^39^ evidence to support a spherical expansion model, at least for moderate refractive errors.

### Spatial Summation Model

**Figure 2.**
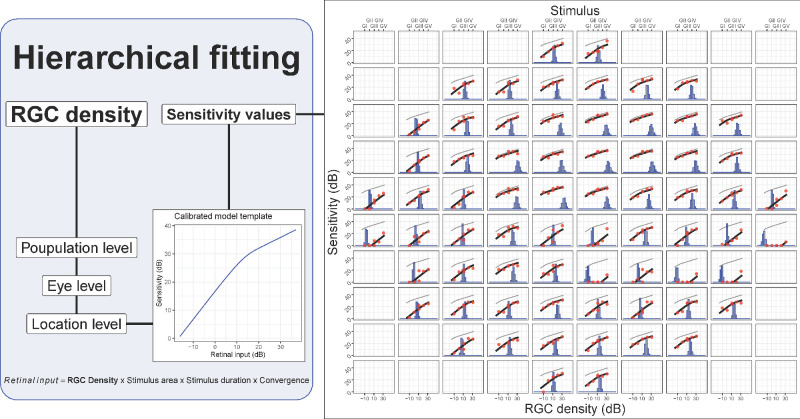
Schematic illustrating the hierarchical fitting process for the template. The template shown on the *left* is shifted horizontally to match the data. The example on the *right* shows the result of the fit. The top horizontal axis reports the stimulus size. The bottom horizontal axis refers to the histograms, which represent the estimated retinal ganglion cell (RGC) density (in dB) for each location. The histograms show all the iterations of the Bayesian fitting procedure. The *red dots* are the measured sensitivity, and the *black lines* are the shifted templates (the original “healthy” template is reported in *light gray*).

A previously described summation model^20^ was used to generate a template to fit the sensitivity versus stimulus area data. The summation model is described in more detail in the Appendix. In brief, the model integrates the total retina input, which is the product of stimulus area, stimulus duration, RGC density, and cone-to-RGC convergence ratio at a specific location. For this application, the stimulus duration was fixed at 200 ms. The model predicts a biphasic relationship between retinal input and sensitivity, with a gradual transition from total to partial summation (Fig. 2). The model accounts for the cone-to-RGC convergence ratio because we found, in previous experiments and calculations,^40^ that the spatial summation response profile (and Ricco's area) did not scale perfectly with the number of RGCs at different eccentricities, but that the number of RGCs needed to be weighted by the number of cones converging onto each RGC. Because different classes of RGCs tile the retina with independent and partially overlapping mosaics, we only consider Parasol (or magnocellular) OFF RGCs (P-OFF-RGCs) for our calculations^41^^,^^42^ because P-RGCs have been shown to be preferentially stimulated by briefly flashed stimuli.^43^^,^^44^ However, for a given location, the effect of stimulus area can be explained by a change in the number of RGCs being stimulated. This indicates a scaling of recruited cortical filters with the amount of total retinal input, at least in healthy observers. Note that we do not attribute any specific role to OFF-RGCs, although a preferential involvement of this subclass of RGCs has been suggested in glaucoma.^45^ This subclass was simply chosen to model a hexagonal mosaic of non-overlapping RGCs^37^^,^^42^ and because OFF-RGCs are the most abundant in the human retina.^46^ Modeling ON-RGCs would have no material effect on our results other than proportionally scaling the underlying RGC density in the model. Structural density of P-OFF-RGCs were obtained as a proportion of the total structural RGC density estimates using the equations provided by Drasdo et al.^34^^,^^37^

In the current study, we wanted to test the hypothesis that such a cortical filter scaling would also occur with RGC damage in glaucoma. This can be done by testing whether the change in sensitivity from RGC damage in glaucoma could be explained by a simple horizontal shift of a summation template predicted by the model, similarly to what was reported by Redmond et al.^16^ This corresponds to a change in Ricco's area (see Fig. 2). To test this hypothesis, we made two assumptions:

1) RGC death and dysfunction would be indistinguishable, meaning that the model would not be able to distinguish whether the reduced input is provided by a smaller number of fully functional cells or a larger amount of dysfunctional cells.^46^
2) The change in sensitivity would be predominantly a consequence of RGC loss and not of photoreceptor damage, media opacity, or other conditions.

An alternative hypothesis was to assume no change in spatial scaling. This corresponds to modeling the change in sensitivity in glaucoma as a vertical shift in the summation template, that is, change in sensitivity without any change in Ricco's area. Note that the actual value of Ricco's area is not reported as part of the results because it is not relevant for testing our hypothesis and because it is not univocally defined for a summation curve with a smooth transition from total to partial summation.

The model template was calibrated with data from the young healthy cohort and tested on patients with glaucoma and age matched controls.

#### Model Calibration

The model has three parameters (see Formula in the Appendix): α determines the vertical offset of the template (in log_10_ scale); τ determines the transition from total to partial summation; and κ determines the slope of the partial summation portion of the curve (slope = 1/κ). The model was calibrated with RGC count estimates and perimetric sensitivity values from the healthy young cohort. The RGC count estimates are more likely to be accurate in this group because of the low likelihood of retinal damage and the close similarity in age with the retinae in the original histology dataset by Curcio and Allen.^35^

The parameters were estimated via numerical optimization (*fminsearch* function in MatLab R2018b, The Mathworks, Natick, MA, USA) and 95% confidence intervals (CIs) for the parameters were computed via bootstrap, resampling individual eyes rather than observations to preserve the correlation structure of the data. The calibrated model was used to generate a template to fit the rest of the data and test our hypothesis, as explained in the next section.

#### Template Fitting to Patients With Glaucoma and Controls

Both the main and alternative hypothesis (spatial scaling versus no spatial scaling in glaucoma) can be tested by fitting the summation template to the perimetric data with different assumptions. Fitting the template presents significant challenges, especially because of the involvement of eyes with advanced damage. The main technical issues are the presence of censored data, because the HFA is not capable of presenting stimuli with luminance greater than 3185 cd/m^2^ (0 dB), and a consequent lack of sensitivity values for more damaged locations. This can, on the one hand, bias the estimates. On the other hand, it makes it difficult to obtain stable estimates for these locations when only few sensitivity values are available at this level of damage. Bayesian computation and hierarchical models can offer a solution because data censoring can be easily incorporated in complex models, avoiding the bias from censored data (i.e. sensitivities < 0 dB), and estimates at individual locations can be made more robust by efficiently distributing information across different levels of the hierarchy.

Details about the implementation of the Bayesian hierarchical model for this study are reported in the Appendix. In brief, for the main hypothesis (spatial scaling), the model estimated the density of RGCs at each location, in log_10_-scale, by optimizing the horizontal shift of the template to fit the observed sensitivity values for each stimulus area (see Fig. 2). The first level of the hierarchy was the population level, modeling the average RGC count. This was then propagated at the eye level and then at each location. The eye and location levels can be considered nested Gaussian random effects. Because of the hierarchical structure, all the data were fitted concomitantly and the estimate at each location was also informed by the data at other locations within the same eye and by the general behavior of the population. The template was used as a link function to model the expected sensitivity at each stimulus area given the modeled RGC density estimate. The response variable was the sensitivity, which was censored at 0 dB. Note that using a link function for the expected sensitivity is different from modeling an inverse transformation of the data. The fitting process also modeled a vertical shift of the template at the population level, to optimize the average centration of the template. The alternative hypothesis (no change in spatial scaling) was implemented with a similar model. In this case, the hierarchical parameter was the vertical shift of the template and the horizontal shift (Ricco's area) was only modeled at the population level. This fitting process assumes no change in spatial scaling across subjects, whereas the change in sensitivity is only modeled through the vertical shift of the template.

Note that it is not possible to model a vertical and a horizontal shift of the template simultaneously, because the solution would be undefined in locations for which the tested stimulus area sizes do not encompass Ricco's area. For example, a location for which all tested stimulus sizes are smaller than Ricco's area can be fitted by arbitrary combinations of vertical and horizontal shifts of the template. Therefore, we used the alternative hypothesis of no spatial scaling as a comparator to assess the significance of our results under the main hypothesis (see the next section). Normally, statistical significance can be assessed by quantifying the uncertainty around parameters’ estimates. However, because each version of the model is forced to fit the data with either a horizontal or a vertical shift of the template, the parameter estimate associated with the modeled shift is likely to be significantly different from zero (no shift) in both cases and cannot be used to accept or reject the tested hypothesis.

#### Data Analysis

All data, including those from the young healthy cohort, were used in the fitting, but only data from the patients with glaucoma and age-similar healthy controls were used to calculate goodness of fit statistics. The R^2^ was calculated for the sensitivity predicted with the template fitted at each location and expressed as the percentage of variance explained. Confidence intervals for the R^2^ were calculated via bootstrap (1000 samples) using the subject as the resampling unit. The root mean squared error (RMSE) was also calculated, for comparison with the structural predictions (see below).

The structure-function analysis was performed in a similar fashion, using the point-wise structural RGC density, calculated as described above, using estimates of GCL thickness from the SD-OCT scans (calculated as the average thickness from the 5 different stimulus sizes). However, because there was no fitting involved in the structure-function predictions, only the RMSE was calculated. Both RGC density estimates were expressed in dB (10*log_10_(Density)). We also calculated the dynamic range for the structural and functional density estimates as the width of the 1% to 99% interval, to report the structural floor effect. All the analyses were performed in R.

When referring to estimates of the total retinal input, we will use the term functional retinal input to refer to the total retinal input calculated with local RGC density values estimated by fitting the functional data. The structural retinal input was instead calculated using structurally derived local RGC density values.

## Results

### Study Population

Descriptive statistics for the sample are reported in Table 1. One individual in the healthy cohort was excluded because they completed only two of the six tests. None of the tests was unreliable.

**Table 1.** Descriptive Statistics of the Sample Reported as Mean (Standard Deviation)

			Glaucoma		
	Healthy < 40 Years Old (*N* = 29)	Age Matched Controls (*N* = 20)	Early (*N* = 10)	Moderate (*N* = 10)	Advanced (*N* = 10)
**Age, y**	28 (3)	62 (11)	66 (9)	59 (10)	62 (11)
**AL (mm)**	24.40 (1.05)	24.00 (0.94)	23.56 (0.65)	24.75 (1.35)	23.71 (1.18)
**24-2 MD (dB)**	−0.67 (0.91)	0.16 (1.36)	−2.26 (1.56)	−8.21 (2.13)	−18.51 (5.78)
**24-2 PSD (dB)**	1.45 (0.37)	1.91 (0.58)	3.24 (1.60)	11.10 (2.35)	11.61 (1.99)
**cpRNFL (µm)**	96.8 (9.2)	93.8 (9.5)	72.0 (10.4)	61.3 (15.4)	47.1 (6.9)
**WRT (µm)**	311.1 (13.8)	303.1 (13.9)	290.5 (17.7)	280.8 (16.2)	275.5 (8.7)
**GCL (µm)**	39.6 (3.10)	37.1 (3.2)	31.8 (4.9)	26.8 (5.4)	23.2 (3.8)
**RGCs (dB)**	5.58 (0.03)	5.54 (0.04)	5.47 (0.08)	5.39 (0.10)	5.32 (0.08)

AL = axial length; MD = mean deviation; PSD = pattern standard deviation; cpRNFL = circumpapillary retinal nerve fiber layer; WRT = whole retinal thickness; GCL = ganglion cell layer; RGCs = retinal ganglion cell count (in 10*log_10_ scale).

The structural metrics are total or average values calculated within the central 10 degrees from the fovea.

### Model Calibration

**Figure 3.**
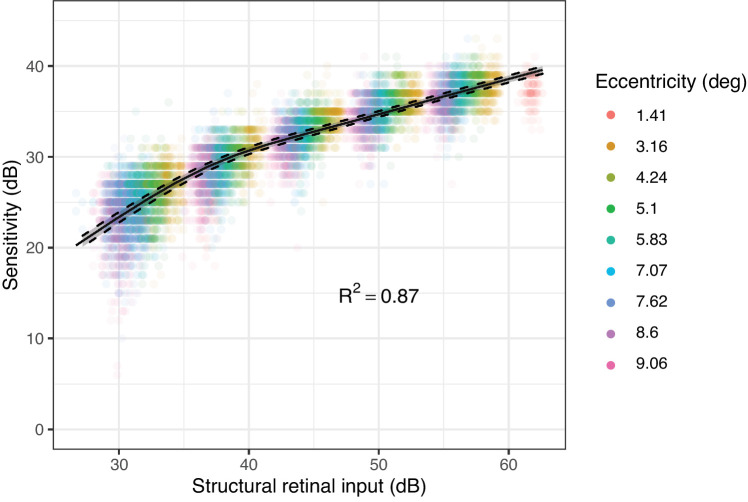
Results of the calibration procedure of the template on the data from the young healthy cohort. The *dashed lines* represent the 2.5% to 97.5% confidence bands for the template estimated via bootstrap. The data are clustered due the different stimulus diameters used.

The parameter estimates for the model fitted in the young healthy cohort were (Mean [95% CIs]): α = 1.42 [95% CI = 1.29, 1.57]; log_10_(τ) = 3.58 [95% CI = 3.44, 3.70]; and κ = 2.59 [95% CI = 2.45, 2.78] (corresponding to a partial summation slope of 0.39 [95% CI = 0.36, 0.40]). The slope was notably different from the commonly chosen 0.25 (*P* < 0.001)^19^^,^^21^ but not dissimilar to the 0.369 reported by Antwi-Boasiako et al. (*P* = 0.146).^17^ The result of the fitting is shown in Figure 3.

### Template Fitting

**Figure 4.**
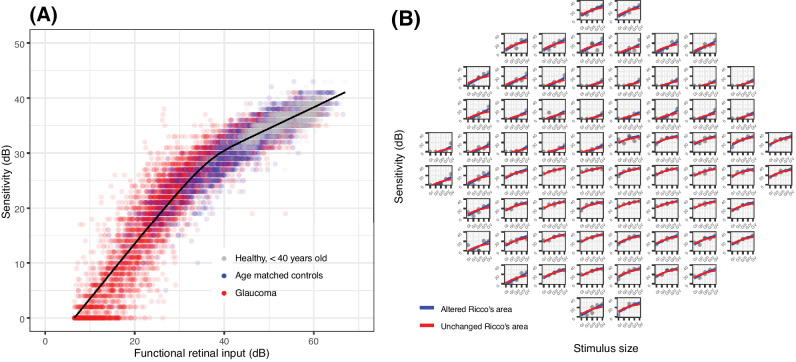
(**A**) Results of the template fitting via horizontal shift on the overall sample. For this graph, the observations from each location were shifted horizontally according to their estimated parasol OFF retinal ganglion cell (RGC) density. (**B**) Example (one eye with glaucoma) comparing the fit obtained via horizontal (altered Ricco's area) and vertical (unchanged Ricco's area) shift of the template.

The horizontal shift of the template (which assumes a change in Ricco's area from RGC damage) explained 95.2% (95% CIs = 94%, 96.2%) of the overall variance in the data, a significant improvement over assuming no change in Ricco's area (*P* < 0.001). Table 2 reports the R^2^ and RMSE values for the healthy subjects and the patients with glaucoma at different stages of damage. Figure 4 shows the fitting results. Supplementary Figure S1 shows the same results for each location (horizontal shift). The average error per subject for the horizontal shift of the template was not significantly affected by age (linear regression, *P* = 0.819), indicating that modeling a change in Ricco's area was able to account for most of the effect of aging. The differences in accuracy between the two alternative models were more evident in the glaucoma cohort with intermediate damage, where a transition from partial to complete summation would be more evident if RGC damage was indeed causing a change in Ricco's area. Supplementary Figure S2 shows the fitting error, stratified by sensitivity, of the two alternative models compared to the test-retest noise. Fitting the template with a horizontal shift produced the closest error to the test-retest noise, consistently below that obtained with a vertical shift.

**Table 2.** R^2^ and Root Mean Squared Error (RMSE) Statistics for the Hierarchical Fitting of the Template

	Estimate [95% CIs]					
	Altered Ricco's Area		Unchanged Ricco's Area		Improvement (%)	
Group	R^2^ (%)	RMSE (dB)	R^2^ (%)	RMSE (dB)	R^2^	RMSE
**All**	95.2 [93.9–96.1]	2.09 [1.92–2.26]	93.2 [91.5–94.5]	2.49 [2.24–2.72]	2.1 [1.6–2.7]	15.9 [12.6–18.3]
**Healthy**	91.3 [90.4–92.1]	1.56 [1.44–1.71]	89.8 [88.8–90.8]	1.69 [1.58–1.83]	1.7 [0.8–2.5]	7.7 [4.0–11.5]
**Glaucoma**						
**Early**	91.6 [89.5–93.1]	2.21 [1.74–2.64]	88.4 [86.6–90.0]	2.59 [1.99–3.10]	3.4 [2.4–4.2]	14.5 [9.70–18.7]
**Moderate**	93.2 [90.9–95.3]	2.96 [2.50–3.39]	89.6 [85.4–93.1]	3.66 [2.98–4.29]	3.9 [2.1–6.2]	19.2 [14.3–22.5]
**Advanced**	95.3 [93.7–96.3]	2.99 [2.70–3.29]	92.3 [89.1–94.3]	3.83 [3.33–4.32]	3.1 [1.9–5.0]	21.8 [17.1–25.2]

The 95% Confidence Intervals were estimated via bootstrap. These statistics exclude the data from the young healthy cohort used for calibration. Improvement was calculated as percent increase in R^2^ and percent reduction in RMSE fitting a horizontal shift of the template over fitting a vertical shift. All improvements were significant (*P* < 0.001).

**Figure 5.**
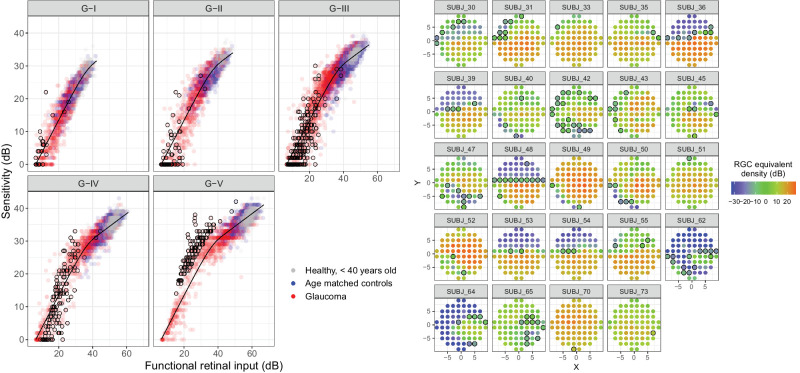
Fitting results split by stimulus size (*left panels*). The observations circled in black are those that exceeded the 97.5% limit of the prediction error for the G-V stimulus. The same locations are reported on the map on the right, representing the modeled RGC density.

When broken down into different stimulus sizes, some locations appeared to have their sensitivity underestimated by the model for the largest stimuli. We identified these locations as those that were greater than 97.5% of the prediction error (4.9 dB) above the prediction with the G-V stimulus (Fig. 5). The sensitivity for these locations also appeared to increase more steeply than predicted by complete summation for smaller stimulus sizes.^47^^,^^48^ We hypothesized that this could be a consequence of testing at the edge of scotomas. When plotted in the 10-2 grid, these locations were in fact mostly located in regions of sharp change in the modeled RGC density estimates (see Fig. 5). We further tested this hypothesis by simulating the response from an RGC mosaic with a sharp change in cell density and we were able to reproduce the same behavior (Supplementary Fig. S3).

### Structure-Function Relationship

**Figure 6.**
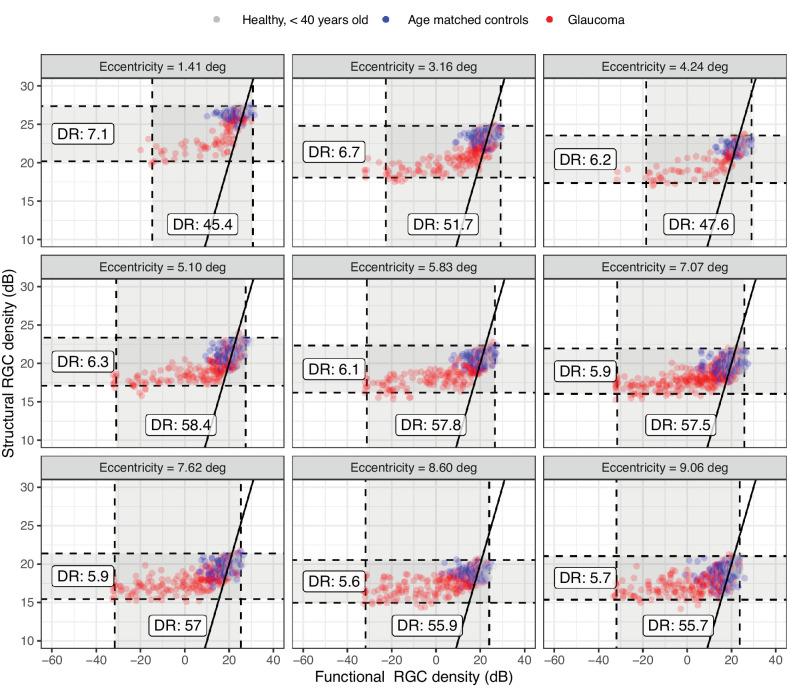
Structural and functional estimates of the Parasol OFF retinal ganglion cell (RGC) density at each location. The *solid line* indicates the identity. The *dashed line* represents the dynamic range (DR) of the structural and functional estimates.

The structural and functional estimates of RGC density are plotted in Figure 6. The overall agreement was poor (Table 3), mostly due to the limited dynamic range of the structural estimates, which was, on average, only 11% (±2%) of the functional estimates.

**Table 3.** Root Mean Squared Error (RMSE) for Structure-Function Predictions

	Structural RMSE (dB) [95% CIs]		
Group	Sensitivity, All Locations	Sensitivity, Locations ≥ 10 dB	Functional RGC Density
**All**	10.6 [8.4–12.5]	3.5 [2.9–3.7]	14.3 [11–17.6]
**Healthy**	3.0 [2.1–3.9]	3.0 [2.2–3.7]	4.0 [2.5–5.3]
**Glaucoma**			
**Early**	5.9 [3.7–7.5]	3.1 [2.4–3.8]	7.2 [4.5–9.3]
**Moderate**	11.8 [9.2–14.1]	4.2 [3.3–4.6]	15.2 [11.6–18.5]
**Advanced**	18.8 [15.7–21.8]	4.8 [3.3–4.9]	26.5 [20.7–31.8]

For sensitivity, structural predictions were generated using the spatial summation template with structural estimates of the parasol OFF retinal ganglion cell (RGC) number as an input. For the RGC density estimates, we report the RMSE of structural estimates of local parasol OFF RGC density predicting the corresponding functional estimates from the fitting of the template.

**Figure 7.**
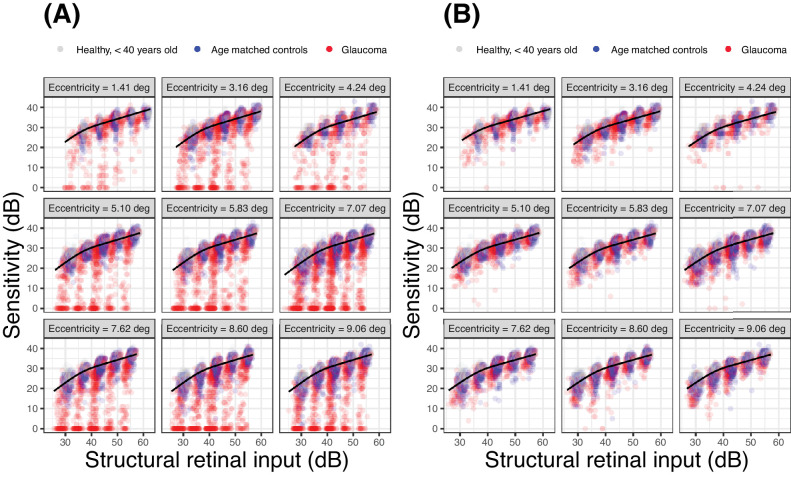
Structure-function predictions based on the template for the whole sample (**A**) and for locations where sensitivity was > 10 dB with a G-I (**B**). The structural retinal input was calculated identically to the functional retinal input, but using structural estimates of local parasol OFF retinal ganglion cell density instead of the functional ones, derived from fitting the template (as in Figs. 4, 5). This is identical to the retinal input calculated for the young healthy cohort for calibration (see Fig. 3), which was also derived from structure.

Using the template to predict the sensitivity from the structural RGC estimates generally provided poor prediction accuracy (see Table 3). These predictions are reported in Figure 7. The predictions were improved, as expected, by only analyzing locations where sensitivity with a G-I stimulus was greater than 10 dB. This latter subanalysis was performed for comparison with the work of Antwi-Boasiako et al.^17^

## Discussion

We evaluated the hypothesis that changes in perimetric sensitivity from modeled RGC damage or loss in the glaucomatous macula could be explained by a change in the spatial scaling of the response of the visual system. We tested this by fitting experimental perimetric data in human observers (patients with glaucoma and healthy controls) with a template that models the relation between stimulus size and perimetric sensitivity. We showed that a horizontal shift of the template, modeling an enlargement of Ricco's area, could explain 95% of the overall variance in the data. This explained the data significantly better than a vertical shift of the template, which would model a change in sensitivity without a change in Ricco's area. We then showed that the local functional loss was not entirely captured by structural measurements from SD-OCT.

Our findings support the hypothesis that RGC damage from glaucoma produces a perimetric functional loss that can be explained by an enlargement of Ricco's area.^16^ This was speculated to be a consequence of the loss of RGCs, leading to the hypothesis that Ricco's area would scale with RGC density, to include a constant number of RGCs. In general, this hypothesis has been shown to hold true in healthy eyes when tested at different eccentricities^8^^–^^14^ and in patients with glaucoma when tested with computational models similar to the one used in this work.^21^ However, Swanson et al.^21^ showed that the extent of Ricco's area depends on the spatial scale of the cortical filters, regardless of the underlying density of RGCs. In fact, previous work has shown that the extent of Ricco's area (and thus the number of RGCs underlying a Ricco's area scaled stimulus^15^) at any given location can be altered, in healthy observers, by stimulation conditions, such as background luminance,^49^^–^^51^ duration of the stimulus,^20^^,^^50^^,^^52^^,^^53^ or by high frequency background noise.^19^ This makes it clear that VF sensitivity cannot be explained solely by RGC density and likely also involves further processing at a cortical level.

Redmond et al.^16^ provided experimental evidence of such a change in the spatial scaling occurring in patients with glaucoma. However, the same phenomenon has not been extensively investigated in advanced glaucomatous damage and in the macular region. Although there is no specific reason to expect spatial summation to behave differently in the macula, its impact would be the greatest in this region for standard perimetry with a G-III stimulus.^8^^–^^10^^,^^21^^,^^30^ This is because the high initial RGC density in the healthy macula would determine a transition between partial and total summation, as RGCs are lost in glaucoma. Moreover, the macula allows direct individualized point-wise structural OCT measurements, which are not usually available for the more peripheral retina. One study by Yoshioka et al.^32^ investigated the effect of spatial summation on the association between perimetric sensitivity and retinal structure in the macula of eyes with early glaucomatous damage and showed that it is improved with smaller stimulus sizes. This is compatible with our findings, because smaller stimulus sizes would operate under complete spatial summation in both healthy and glaucomatous eyes, making the slope of the relationship between the number of RGCs and sensitivity steeper. One important difference was the method used to displace the stimuli to account for RGC displacement, which, in the case of Yoshioka et al.,^32^ was later shown to yield less accurate results, especially in the parafoveal region.^34^ This was then also confirmed by the same group in later work.^54^ More recently, a detailed analysis has been presented by Antwi-Boasiako et al.,^17^ who studied the relationship between macular RGC counts and perimetric sensitivity in nonhuman primates with experimental glaucoma. Antwi-Boasiako et al.^17^ also analyzed their data within the framework of spatial summation. Some of their results were confirmed in our study. Importantly, the partial summation slope estimated by our data (0.39, corresponding to an exponent κ of 2.59) was very close to their estimate (0.369). This is noteworthy, because there is still uncertainty about the most accurate choice of slope to describe partial summation for perimetric stimuli in studies of this kind. In computational models of sensitivity, this mainly depends on the choice of the spatial filter and of the Minkowski summation exponent κ.^19^ Common choices for the exponent are between 2 and 4. For most symmetric filter choices (except some Gaussian derivatives used to model cortical responses), these values correspond to a partial summation slope of 0.5 (Piper's law) and 0.25. An exponent of 4 was used in a previous implementation of our model^20^ and by others.^19^^,^^21^ However, an intermediate value for the exponent seems more reasonable given the experimental results from this work and Antwi-Boasiako et al.^17^

Differently from Antwi-Boasiako et al.,^17^ we found that structural measurements were not able to fully characterize functional damage, owing to their reduced dynamic range (see Fig. 6). One factor that could explain this discrepancy is that Antwi-Boasiako et al.^17^ had access to histology-derived RGC counts in both healthy and glaucomatous eyes to calibrate their structural models, which would naturally improve accuracy. In contrast, we only relied on limited histology data in healthy human subjects provided by Curcio and Allen.^35^ Additionally, it is unclear from their paper whether Antwi-Boasiako et al.^17^ accounted for RGC displacement by simply moving the center of the 10-2 stimuli, as in Yoshioka et al.,^32^ or whether they applied the displacement to the edge of the stimulus (see Fig. 1). This is relevant because, despite yielding correct RGC counts in healthy eyes and in early damage, our method of displacement, by its nature, amplifies the floor-effect, because non-functional residual tissue is summed over a larger area, especially in the parafovea. Finally, the level of damage in Antwi-Boasiako et al. was in general less advanced than in our dataset, with the lowest sensitivity values being approximately 10 dB. Indeed, restricting our analysis to locations with a sensitivity > 10 dB with a G-I stimulus resulted in a great improvement in the RMSE for structure-function estimates (see Table 3, Fig. 7). Nevertheless, our results find ample confirmation in previous literature^36^^,^^55^^,^^56^ documenting a structural floor-effect at around 10 dB of sensitivity loss in the macula and confirming that structurally derived estimates offer only a partial description of RGC loss and damage occurring in glaucoma. All these aspects, including the increased level of perimetric noise at more advanced damage, contributed to the poor RMSE in the structure-function predictions reported in Table 3.

Our findings have important implications for the interpretation of macular perimetric damage in glaucoma. The first important aspect is that it confirms a change in the spatial scale of the response following RGC loss or damage, which corresponds to an enlargement of Ricco's area. As previously stated, the exact value of Ricco's area is irrelevant for testing our hypothesis and is not univocally defined for curves with a smooth transition from total to partial summation. However, Ricco's area is a useful concept to describe changes in spatial scaling, and here it is used as synonymous of spatial scale. One thing that should be noted is that previous work mostly focused on the relationship between the number of RGC receptive fields covered by the stimulus and perimetric response. According to this view, the response of the visual system would scale to include a constant number of RGCs at Ricco's area.^16^^,^^17^ Our interpretation differs slightly, because the total retinal input in our summation model would not differentiate between reduced input from RGC loss or dysfunction. Differentiating between these two contributions would require additional investigations. Adaptive optics OCT imaging has shown promising results allowing direct visualization of RGCs in healthy subjects^57^ and patients with glaucoma^58^ and could be used to more precisely quantify the density of RGCs. Functional tests, such as high contrast grating stimuli, could be used for the same scope.^46^^,^^59^^–^^62^

The varying relationship between RGC damage and functional loss is especially important in the macular region, because sensitivity to the widely used G-III, 200 ms stimulus would initially be determined by partial summation, making the relationship with retinal structure shallow. As RGCs are lost or damaged, the response would gradually transition into complete summation, where the relationship between sensitivity and retinal structure becomes steeper. This implies that, for the same percentage of RGC loss, changes in sensitivity would be much smaller early in the disease compared to more advanced damage. This might make the detection of early damage, and similarly early progression, more challenging.^6^^,^^63^ Other strategies using smaller targets or shorter durations for macular stimuli might make perimetric tests more efficient by testing always under complete summation conditions, although this might limit the dynamic range of the test. Some of these strategies have already been adopted in some home monitoring devices.^64^ Another approach would be to modulate the area or duration of the target instead of their luminance. This approach would take full advantage of the horizontal translation of the response profile observed in our data and in previous publications,^16^^,^^63^ effectively testing the response at a fixed point of the summation curve. Such an approach has been shown to maximize signal-to-noise ratio in glaucoma and to reduce response variability compared to luminance modulation.^63^

It should be noted that, whereas fitting a template and testing the spatial-scaling hypothesis did not require a link to RGC density, modeling the retinal input and the effect of RGC loss provides a linkage to an underlying biological substrate, offering a generalizable framework for interpreting the results. For example, using a computational model of an RGC mosaic allowed us to provide a possible explanation for the edge effect for larger perimetric stimuli observed in the data (see Supplementary Material). Moreover, modeling changes in retinal input rather than simple translations of “healthy” summation functions for each tested location highlighted how changes in spatial summation both across the healthy VF and as a consequence of damage can arise in the context of different modifications to the same underlying biological substrate. It should finally be highlighted that, because of how the spatial summation template was calculated (i.e. using sensitivity values and estimated RGC counts in healthy subjects), the intrinsic linkage to the underlying retinal input is present in our calculations, regardless of whether it is made explicit or not in our interpretation of the results.

A better characterization of the relationship between RGC damage and perimetric sensitivity is also useful to improve the correspondence between perimetric changes and structural damage observed with imaging. As shown in this and previous work,^32^^,^^36^ both measurements can be reported in a log-scale of RGC number. This could facilitate structure-function analyses for progression or enable seamless integration of structurally derived metrics into perimetric strategies.^65^ One limitation, however, is that structural metrics do not seem to have enough dynamic range, at least locally, to capture the full extent of functional damage measured by perimetry. Although such a discrepancy has been reduced by nonlinear estimates, such as with help of artificial intelligence,^66^^–^^68^ structural tests are unlikely to replace perimetry. An efficient integration of the two sources of information seems, therefore, the most effective way of diagnosing and monitoring glaucoma.

A limitation of this work is that it was not possible to derive sensitivity estimates for all stimulus areas at all tested locations, especially among patients with intermediate or advanced glaucoma. This was expected given the technical limitations of the device (limited stimulus areas and fixed duration), and addressed with the use of a hierarchical model, which allowed for more robust estimates of RGC damage for locations where only limited data could be collected, and by accounting for censoring at 0 dB. However, the estimates for these locations are necessarily less precise and mostly reliant on the behavior of the other locations within the same eye and on the general trend of the overall population. For the same reason, it was not possible for us to model the horizontal and vertical shift at the same time, because the fitting results would only be fully constrained for locations that span both partial and complete summation with the available stimulus diameters. For example, for locations exhibiting complete summation exclusively, the same fitting result can be achieved by either a vertical or a horizontal translation of the template. However, this would not affect the ability to compare our two alternative hypotheses. It is also important to note that previous work, especially by Gardiner et al.,^69^^,^^70^ has shown poor correlation between accurate sensitivity estimates derived from frequency of seen curves and clinical perimetry, especially for values < 20 dB. In our analysis, however, we assumed that low sensitivities would still provide useful information to test population-level hypotheses, especially in eyes with advanced glaucoma. We provide, as supplementary, additional analyses supporting this assumption. Importantly, we show that including sensitivity values ≤ 15 dB reduced the prediction error for the fitted model for sensitivity values > 15 dB. This indicates that, in our data, locations with advanced damage improved the precision of the model.

In our study, we could not control for the effect of optics on macular sensitivity. This could have been influenced by age-related changes to refractive media. We controlled for this limitation by comparing glaucoma with age similar controls. The effect of optics^71^^,^^72^ and aging^73^ on spatial summation is still unclear. Redmond et al.^73^ did not find any change in the critical area with age. However, from our data, there does not seem to be any significant residual effect of aging on explaining the change in sensitivity once the change in spatial summation is accounted for. However, our data do not allow us to test this hypothesis specifically and, further, more targeted investigations, are needed.

## Supplementary Material

Supplement 1

## Appendix

### Computational Model

The model, as previously explained,^20^ predicts sensitivity as a function of the total retinal input, which is the product of the number of RGC receptive fields that underlie the stimulus, the duration of the stimulus presentation, and the cone-to-RGC convergence ratio at different eccentricities. This was derived by combining Curcio and Allen's data^35^^,^^74^ and the RGC receptive field (RGC-RF) density obtained from the equations provided by Drasdo et al.^34^^,^^37^ In our previous analysis of spatial summation data in healthy subjects,^20^ we showed that this weighted RGC-RF number, rather than the raw count of RGC-RFs covered by the stimulus, were able to equate the spatial summation curves at different eccentricities. The model uses a capacitor equation and continuous integration over the input. A Minkowski exponent is used in the integration, similar to the vector summation equation used by Pan and Swanson.^19^ The model has three parameters that can be fitted: α determines the vertical offset of the template (in log_10_ scale); τ determines the transition from total to partial summation; and κ determines the slope of the partial summation portion of the curve (slope = 1/κ). The formula from Montesano et al.,^20^ with small modifications, is reported below:

$$\begin{array}{c} R=10^{\alpha }{\left(\int_{0}^{T}M^{k}d\left(st\right)\right)}^{1/k} \end{array}$$

where R is the sensitivity in linear units (10^dB/10^), M is the total retinal input filtered (convolved) with a capacitor equation in the form

$$\begin{array}{c} M=\exp\left(-st/\tau \right)\;*\;S \end{array}$$

where S is a step function of the retinal input and is equal to 1 over a segment of *st* (an arbitrary unit of spatio-temporal input) that indicates the extent of the total retinal input of the stimulus, that is, it becomes longer when more RGCs are stimulated or the same RGCs are stimulated for a longer period of time. The symbol * indicates the convolution operation.

### Bayesian Fitting

The fitting sought to find the optimal value of RGC density for each location that would give the best fit for the template. Changing RGC density corresponds to a horizontal shift of template. RGC density at each location was modelled as a hierarchical random effect, nested within another random effect grouping locations from the same eye. A single global parameter also allowed a vertical offset of the template to achieve the optimal fit in the overall sample. This offset was however very small (−0.23 dB). The same procedure was adopted to fit vertical shifts of the template at each location (i.e. no change in Ricco's area), while a global parameter optimized the location of Ricco's area in the whole sample (this offset was also small, −0.05 log_10_-units). Note that the template was not allowed to move both horizontally and vertically at each eye/location because this would make the fitting undetermined for all locations where sensitivity values showed no change in slope in the data, because the same fit could be obtained by infinite combinations of vertical and horizontal shifts.

VF sensitivity was assumed to have a normal distribution of the residuals, censored at 0 dB. Fitting of the Bayesian model was achieved using JAGS (Just Another Gibbs Sampler^75^) to run Markov Chain Monte Carlo (MCMC) simulations, within the R environment (R Foundation for Statistical Computing). Two parallel MCMCs were run for at least 5000 iterations after 1000 adaptation steps and 5000 burn-in iterations. The MCMCs were stopped if the Gelman-Rubin diagnostic was < 1.2 for all the monitored parameters, indicating convergence.^76^ Prior distributions on the fixed effects were non-informative normal distributions with a precision of 0.01 (variance = 100).
