# Supplementary material for: Spatial Summation in the Glaucomatous Macula: A Link With Retinal Ganglion Cell Damage
Source: Invest Ophthalmol Vis Sci. 2023 Nov 27;64(14):36. doi: 10.1167/iovs.64.14.36 (PMC10683773; doi:10.1167/iovs.64.14.36)
Supplement: Supplement 1 [file iovs-64-14-36_s001.pdf]

Supplementary to: “Spatial summation in the glaucomatous macula: a link with ganglion cell damage”

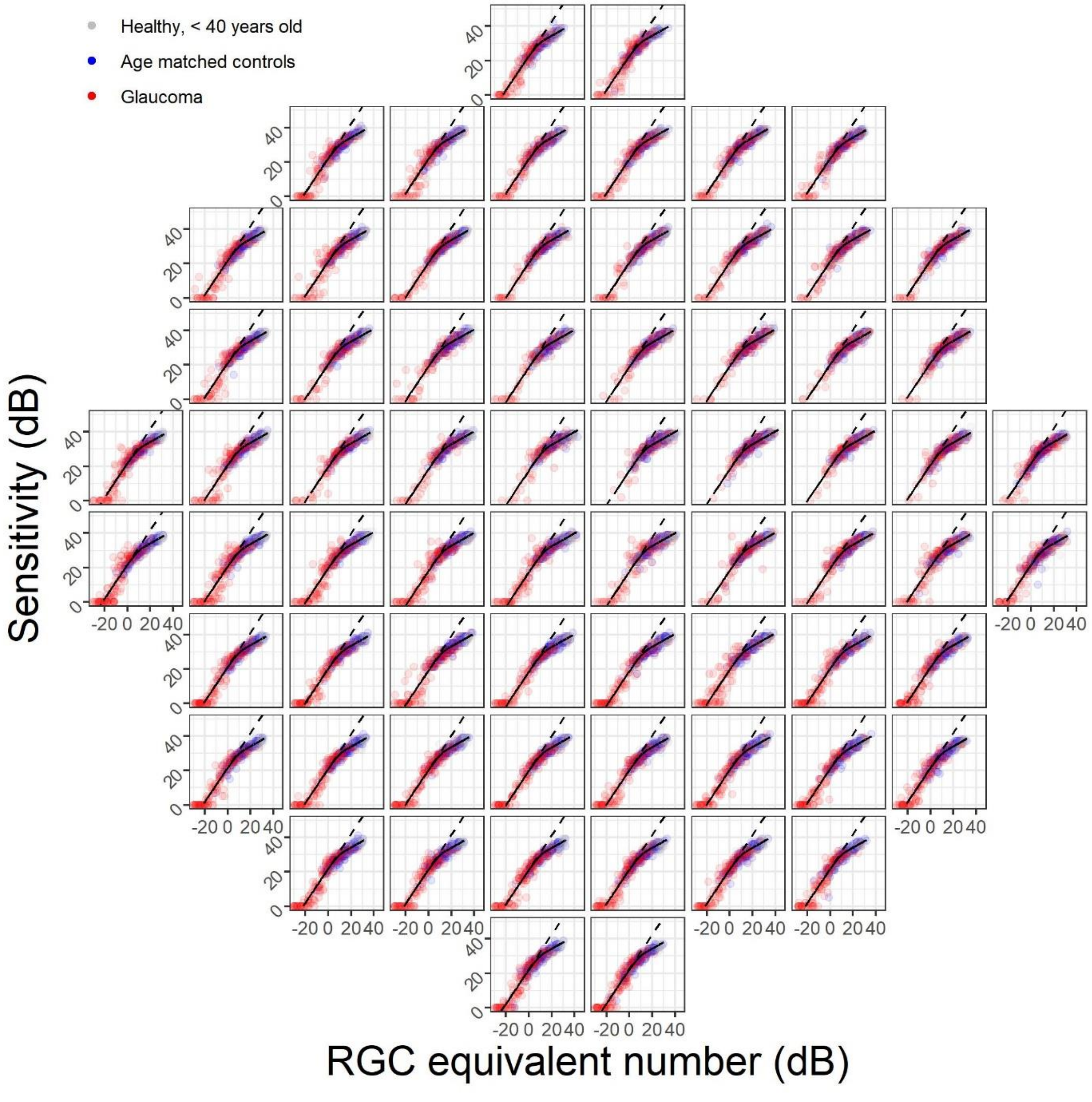

Supplementary Figure 1. Template fit for each location of the 10-2 grid. The dashed lines indicate total summation. The horizontal axis reports the RGC equivalent count, i.e. the effective RGC contribution to the response as a combination of a RGC loss and dysfunction. This representation is possible because each location is plotted separately and the effect of Cone:RGC convergence can be accounted for in each plot, leaving the RGC density as the only varying factor in each subplot. RGC = Retinal Ganglion Cells.

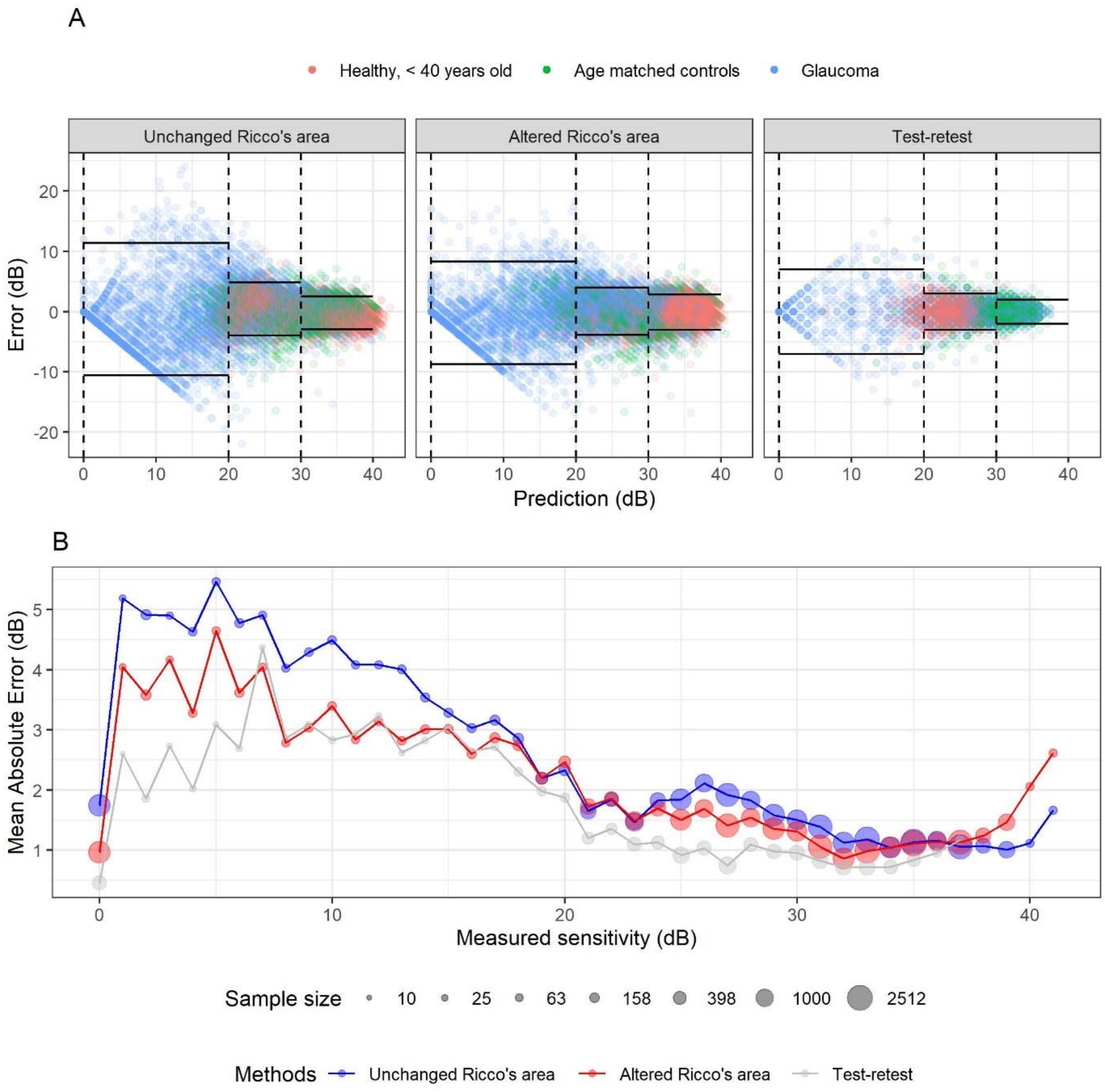

Supplementary Figure 2. A) Prediction against error plots for the two fitting procedures, i.e. horizontal shift (altered Ricco's area) and vertical shift (unchanged Ricco's area), compared to the test-retest variability. For test retest, the G-I stimulus was used for the healthy young cohort and the G-III stimulus was used for the glaucoma cohort and the age matched controls. The "prediction" for test-retest was calculated as the average between the two test repeats. The error was the difference of each repeat from their average. The solid lines represent the 95% limits of the error, calculated separately for three sensitivity levels. **B)** Absolute error stratified by measured sensitivity for the tests-retest and the prediction from the template. The best estimate of sensitivity used to calculate the error and to stratify the plot is the average of two tests repeats and the prediction from the template, respectively.

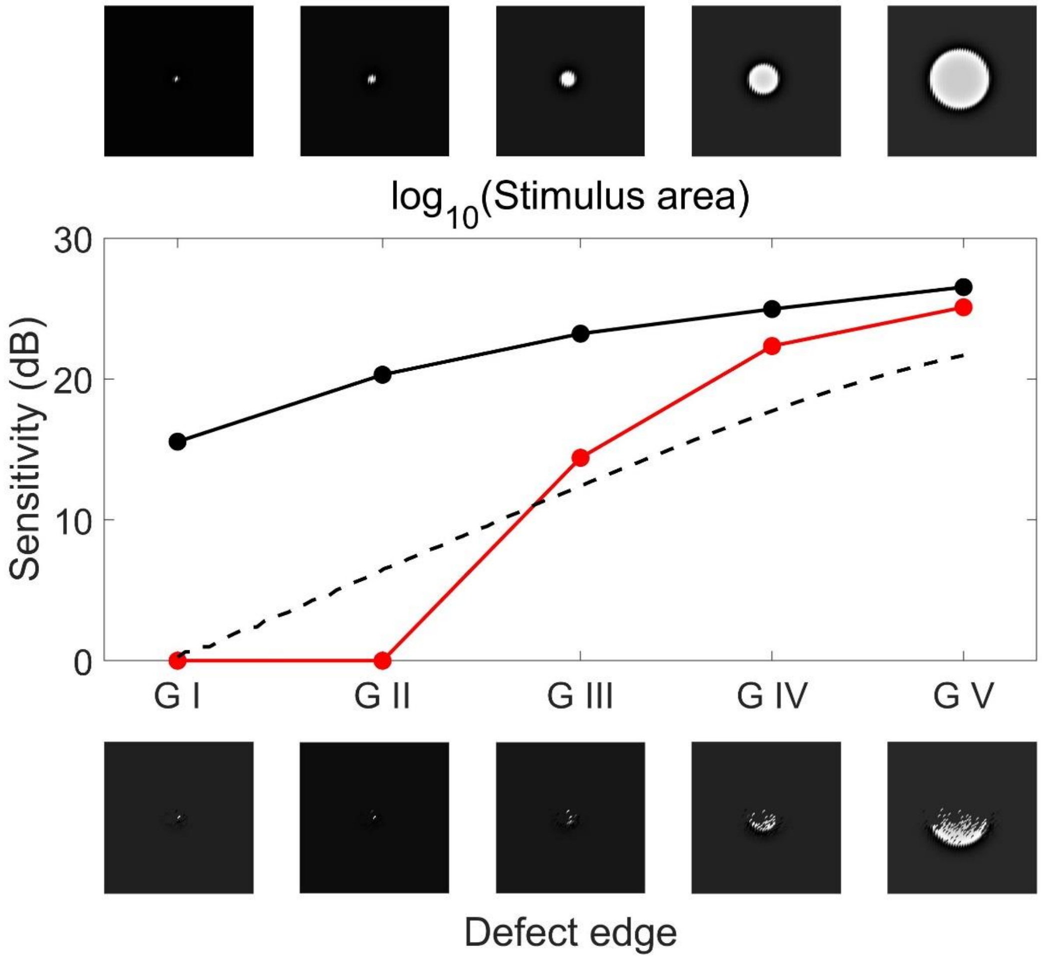

Supplementary Figure 3. Example of how a sharp edge can produce the deviation from the model observed in the data. These responses are calculated from a full computation model simulating an RGC mosaic. The healthy mosaic is reported at the top (and the corresponding response is in black, solid line). The degraded mosaic with a sharp edge is reported at the bottom (and the corresponding response is in red). The dashed line represents the best fit of the template to the data generated from the degraded mosaic. Note how a sharp edge introduces deviations from the template, which assumes a homogeneous RGC density in the tested area. RGC = Retinal Ganglion Cell.

Influence of low perimetric accuracy for advanced damage

Gardiner et al.[11, 12] showed that estimates of sensitivity obtained with SITA algorithms correlated poorly, for low sensitivity, with accurate estimates of the 50% threshold measured with frequency-of-seen curves, demonstrating a ‘floor effect’. The level of this floor is usually placed between 15 dB and 20 dB (note that the 10 dB floor for our analysis was chosen for comparison with Antwi-Boasiako et al.[1]). While this issue certainly affects estimates at the level of individual locations or eyes, we hypothesised that low sensitivity values would still provide useful information for population level estimates. We have performed two additional analyses, reported below, to confirm that this is the case.

We first tried to replicate, via simulations, the results that would be obtained from the full-threshold (FT) strategy implemented on the Humphrey Field Analyzer (HFA). While the specific details are not known, the Open Perimetry Interface (OPI)[13] offers an implementation of the FT strategy based on the best available knowledge[14]. We also tried to replicate the HFA 'growth-pattern' approach for a 10-2 grid based on what is known about the 24-2 grid[14]. Briefly, the seed points were the locations at coordinates {±3; ±3}. Each quadrant was treated independently. The testing sequence progressively extended to the periphery in three concentric clusters around the seed points. The FT 4-2 staircase started at the expected normative value for a G-III stimulus at the seed points and, for the other locations, at the average sensitivity of their nearest neighbours for which sensitivity had already been determined.

The ground-truth for the simulations were the thresholds predicted by the spatial summation functions fitted under H1 (changing Ricco's area) on the original data. The objective was to see whether it was possible to retrieve the original 'ground-truth' RGC density by fitting the model under H1 to the simulated data. If the testing strategy introduced a floor effect, we would observe a proportional bias in our estimates. The responses were simulated using the formula provided by Henson et al.[15] for response variability, capping the standard deviation of the Gaussian psychometric function at 10 dB[16]. The estimated and ground-truth RGC density values are reported in **Supplementary Figure 4**. While there was a consistent offset, there was no proportional bias, indicating no floor effect from the testing strategy used for our study. Interestingly, the consistent offset was due to an underestimation of higher sensitivities (see **Supplementary Figure 4**). However, these results can vary based on the starting values of the FT strategy. These were set to the normative G-III sensitivity for the four initial seeding points in our simulations, but we cannot be sure of what starting points are being used in the HFA for other stimulus sizes.

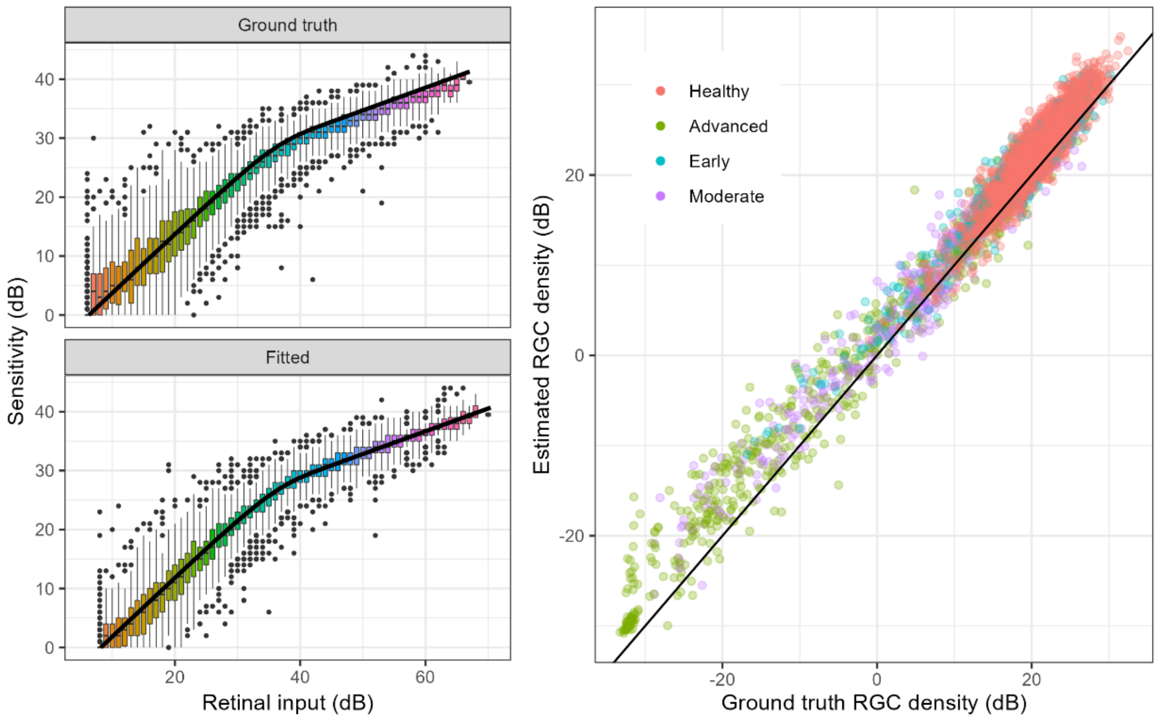

Supplementary Figure 4. The left panels show a comparison of the summation model with the results of the simulations. The sensitivity values in the box-plots were grouped by ground-truth (top) or fitted (bottom) retinal input values, in rounded decibel units. Note how the results of the simulated full-threshold strategy for

high sensitivity values are slightly lower than the ground-truth sensitivity, indicated by the black line. The fitting procedure accounted for that by estimating a retinal ganglion cell density higher than the ground truth. This is shown in the right panel, where the diagonal indicates identity. This produced a better fit to the data (left bottom panel). Note that this is similar to shifting the model template (black line) down and to the left in the top left plot.

In our second analysis, we fitted the data under H1 censoring the sensitivity values at 15 dB instead of 0 dB (as in the original analysis). Note that the model would still retain the information that these values are smaller than 15 dB. We compared the Root Mean Squared Error (RMSE) and R^2 for the predictions obtained with the parameters fitted with the two levels of censoring ($RMSE_0$ and $RMSE_{15}$ respectively). Importantly, both the predictions and the data for this comparison were floored at 15 dB, regardless of the censoring level used for fitting. This ensured a fair comparison. Confidence intervals were calculated via bootstrap, as in the main analysis.

This analysis could have had three possible outcomes:

1. $RMSE_{15}$ was not different from $RMSE_0$: this result would indicate that sensitivity values below 15 dB provided no additional information to increase the prediction accuracy for sensitivities above 15 dB.
2. $RMSE_{15}$ was better (smaller) than $RMSE_0$: this would indicate the presence of spurious information in sensitivities 15 dB with a detrimental effect on the accuracy of the estimates. This would be the strongest indication of a floor effect biasing the estimates.
3. $RMSE_{15}$ was worse (larger) than $RMSE_0$: this would indicate that sensitivity values below 15 dB provided useful information to increase the accuracy of the estimates above 15 dB.

The results are reported in the **Summary Table 1** below. $RMSE_0$ was significantly better than $RMSE_{15}$, a clear indication that locations with advanced damage can provide useful information for the accuracy of the estimates. Interestingly, the differences in RMSE were even more prominent in advanced cases.

Group		Estimate [95%-CIs]					
		Censored at 15 dB		Censored at 0 dB		Improvement (%)	
		R^2 (%)	RMSE (dB)	R^2 (%)	RMSE (dB)	R^2	RMSE
**All**		91.0 [89.7-92.0]	2.04 [1.87-2.22]	93.5 [92.5-94.3]	1.74 [1.64-1.85]	2.7 [2.0-3.3]	14.9 [11.8-17.6]
**Healthy**		90.8 [89.5-91.8]	1.59 [1.45-1.76]	91.4 [90.4-92.3]	1.53 [1.42-1.67]	0.7 [0.4-1.1]	3.6 [2.2-4.9]
**Glaucoma**	**Early**	88.0 [85.6-89.7]	2.15 [1.84-2.49]	91.6 [89.8-93.0]	1.80 [1.57-2.06]	3.9 [2.7-5.2]	16.2 [11.6-20.6]
	**Moderate**	86.2 [80.7-90.6]	2.80 [2.30-3.20]	91.8 [89.5-93.9]	2.15 [1.88-2.39]	6.1 [3.1-10.1]	23.0 [16.3-28.8]
	**Advanced**	86.7 [81.5-90.0]	2.85 [2.52-3.21]	92.6 [90.0-94.3]	2.13 [1.89-2.37]	6.4 [4.5-9.6]	25.4 [23.0-27.5]

Supplementary Table 1. Prediction error from the same model (horizontal translation) fitted by censoring data at 0 dB (original) or at 15 dB. The prediction error for both was evaluated by capping the sensitivity values at 15 dB (i.e. all values smaller than this threshold were set to 15 dB both for the data and predictions). The 95%-Confidence Intervals were estimated via bootstrap. These statistics exclude the data from the young healthy cohort used for calibration. Improvement was calculated as percent increase in R^2 and percent reduction in RMSE. All improvements were significant ($p < 0.001$). RMSE = Root Mean Squared Error.
